# GETTING OVER THE FIRST HURDLE: PREPARING YOUR MANUSCRIPT FOR A POSITIVE REVIEW

**DOI:** 10.1093/geroni/igz038.821

**Published:** 2019-11-08

**Authors:** Suzanne Meeks

**Affiliations:** University of Louisville, Louisville, Kentucky, United States

## Abstract

In this section of the symposium, I will talk about manuscript preparation for maximizing the likelihood that your work will be sent for review. I will talk about common author errors that usually guarantee an immediate reject decision such as not reading and following the Instructions to Authors. I will emphasize the importance of plain, good writing. Editors of high impact journals receive 10 or more new manuscripts per week, and due to limited page space, have to reject 80-90% of them. Regardless of scholarly quality, if the point and contribution are not clear in a quick scan of the paper, it likely will not be reviewed favorably. I will provide tips for strong scientific writing that are commonly violated in manuscript submissions, and provide references for additional writing support.

